# A deep learning model (FociRad) for automated detection of γ-H2AX foci and radiation dose estimation

**DOI:** 10.1038/s41598-022-09180-2

**Published:** 2022-04-01

**Authors:** Rujira Wanotayan, Khaisang Chousangsuntorn, Phasit Petisiwaveth, Thunchanok Anuttra, Waritsara Lertchanyaphan, Tanwiwat Jaikuna, Kulachart Jangpatarapongsa, Pimpon Uttayarat, Teerawat Tongloy, Chousak Chousangsuntorn, Siridech Boonsang

**Affiliations:** 1 Department of Radiological Technology, Faculty of Medical Technology, Mahidol University, Nakhon Pathom, Thailand; 2 Division of Radiation Oncology, Department of Radiology, Faculty of Medicine Siriraj Hospital, Mahidol University, Bangkok, Thailand; 3 Center for Research and Innovation, Faculty of Medical Technology, Mahidol University, Nakhon Pathom, Thailand; 4 Nuclear Technology Research and Development Center, Thailand Institute of Nuclear Technology (Public Organization), Nakhon Nayok, Thailand; 5 Center of Industrial Robot and Automation (CiRA), College of Advanced Manufacturing Innovation, King Mongkut’s Institute of Technology Ladkrabang, Bangkok, Thailand; 6 Department of Electrical Engineering, School of Engineering, Faculty of Engineering, King Mongkut’s Institute of Technology Ladkrabang, Bangkok, Thailand

**Keywords:** Biomarkers, Software, Computer science

## Abstract

DNA double-strand breaks (DSBs) are the most lethal form of damage to cells from irradiation. γ-H2AX (phosphorylated form of H2AX histone variant) has become one of the most reliable and sensitive biomarkers of DNA DSBs. However, the γ-H2AX foci assay still has limitations in the time consumed for manual scoring and possible variability between scorers. This study proposed a novel automated foci scoring method using a deep convolutional neural network based on a You-Only-Look-Once (YOLO) algorithm to quantify γ-H2AX foci in peripheral blood samples. FociRad, a two-stage deep learning approach, consisted of mononuclear cell (MNC) and γ-H2AX foci detections. Whole blood samples were irradiated with X-rays from a 6 MV linear accelerator at 1, 2, 4 or 6 Gy. Images were captured using confocal microscopy. Then, dose–response calibration curves were established and implemented with unseen dataset. The results of the FociRad model were comparable with manual scoring. MNC detection yielded 96.6% accuracy, 96.7% sensitivity and 96.5% specificity. γ-H2AX foci detection showed very good F1 scores (> 0.9). Implementation of calibration curve in the range of 0–4 Gy gave mean absolute difference of estimated doses less than 1 Gy compared to actual doses. In addition, the evaluation times of FociRad were very short (< 0.5 min per 100 images), while the time for manual scoring increased with the number of foci. In conclusion, FociRad was the first automated foci scoring method to use a YOLO algorithm with high detection performance and fast evaluation time, which opens the door for large-scale applications in radiation triage.

## Introduction

Upon exposure to ionizing radiation, several types of DNA damage occur including base damage, single- and double-strand breaks^1^. Of these, DNA double-strand breaks (DSBs) are the most lethal to the cells. Several techniques have been used to quantify the amount of DNA DSBs, such as comet assay^2,3^, radiation-induced foci assay^4^, pulsed-field gel electrophoresis^5^. DNA damage and repair proteins come into play after damage is caused following exposure to ionizing radiation. One of the signaling responses includes the phosphorylation of histone H2AX at serine 139 (γ-H2AX) within the DNA damage site when DSBs are formed^6^. ATM and DNA-PK play important roles in the phosphorylation of γ-H2AX^7–10^. γ-H2AX has become a potential biomarker of DNA DSBs (which increase in proportion to radiation dose)^11^. Following the formation of DNA damage, several repair proteins (such as those involved in homologous recombination and nonhomologous end joining pathways) are recruited to the damage sites to repair the broken ends^12,13^. If DNA DSBs are left unrepaired, they can lead to mutagenesis, disease and cancer^14^. γ-H2AX quantification to determine the amount of DNA DSBs were applied in nuclear medicine^15,16^, radiotherapy monitoring^17–19^, diagnostic monitoring^20–22^, genotoxicity testing^23^ and biological dosimetry (biodosimetry)^24–26^.

To assess the radiation dose received by a population where physical dosimetry is not available, several biodosimetry methods can be applied such as dicentric, micronucleus, premature chromosome condensation, fluorescence in-situ hybridization (FISH) translocation and γ-H2AX assays^27^. The dicentric assay has been the gold standard for cytogenetic assessment. However, the dicentric assay requires several days for cell culture and assessment by trained personnel^28^. To date, the γ-H2AX assay has been widely used as a rapid and sensitive technique to assess radiation exposure^29^. The quantification of γ-H2AX can be performed by Western blot, flow cytometry or foci counting^30,31^. The quantified γ-H2AX values are interpolated onto an established dose–response calibration curve to estimate the received dose in an individual. The number of γ-H2AX foci is typically quantified directly by eye. Manual γ-H2AX foci scoring relies on time-consuming and tedious work, and it is prone to human bias^32–35^, however it is still the most accurate and hence is preferred by many biodosimetry laboratories^36^.

In recent decades, automated and semi-automated foci scoring approaches (based on image processing algorithms, such as TGI^37^, FociCounter^38^, AutoRIF^39^, AKLIDES®^34^, FoCo^40^, Focinator^41,42^, AutoFoci^43^, FocAn^44^ and others^33,45–48^) have used multiple object identification algorithms to segment nuclei and allow discrimination of γ-H2AX foci. These image processing algorithms utilize various techniques to distinguish foci, such as Otsu’s thresholding^49^, top-hat transformation^37,47,48,50^, H-Dome/threshold-based segmentation^37^, pattern recognition^51^. Some studies^37,44^ also developed extended approaches to separate the overlapped foci which occur in nuclei with high DSB density^33,39,48,52,53^. In addition, FocAn^44^ can be used as an automated 3-dimensional analysis of γ-H2AX foci to overcome the limitation of 2-dimentional counting. However, automated foci identification software limited the option for users to train the detection algorithms based on their data and match them with their specific tasks^32^.

In microscopy, machine learning has been widely used to develop predictive tools for biomedical applications^54–58^. For foci identification and quantification, machine learning was introduced by *Herbert *et al*.* (2014) to train a new foci detection algorithm (called ‘FindFoci’) and provided it as an open-source plugin for ImageJ^32^ (and obtained an F1 score > 0.9). However, they studied Msh4-GFP and Zip3-GFP foci in 21 images with little variation of noise and artefacts. In 2019, *Gu *et al.^59^ proposed a machine learning-based linear support vector machine classifier combined with real-time image processing for sorting cells based on the count of γ-H2AX foci in human glioblastoma cells treated with 6 Gy gamma irradiation and reported a good correlation (R^2^ = 0.881). In 2020, *Hohmann *et al*.*^60^ published their examination of multiple machine-learning models [multi-layer perceptron (MLP) classifier, support vector machine, complement naive Bayes classifier and random forest classifier] to classify γ-H2AX foci in fluorescently labeled images containing multiple types of artefacts. Three glioma cell lines (U-251 MG, LN-229 and U-343 MG) irradiated with 2 or 4 Gy were used for training their model. MLP produced the best results, with an F1 Score = 0.923. However, the challenge of foci overlapping due to clustering remained unsolved.

Deep learning based convolutional neural network (CNN) is proposed for cell biology imaging. *CellProfiler3.0*^61^ software has been configured to make use of cutting-edge CNNs to analyze biomedical images by introducing support modules for deep learning system-based TensorFlow^62^ or Convolutional Architecture for Fast Feature Embedding (Caffe)^63^. The ‘ClassifyPixels-Unet’ module is used for nuclei segmentation using Unet CNN^64^. ‘MeasureImageFocus’ module is used to train a deep learning model^65^ to detect foci in images. In 2020, *Chen and colleagues*^66^ proposed a deep learning-based open-source pipeline (called ‘FociNet’) which used U-Net CNN^64^ for Hela-EGFP-53BP1 cell segmentation and a VGG-19-based classification network^67^ for classifying a single cell as normal, damaged or nonsignaling. The accuracy of the classification network reached 99.15% in the validation data set. In the same year, *Vicar *et al*.*^68^ proposed a deep learning-based fully-automated method for 53BP1 and γ-H2AX foci counting called ‘DeepFoci’ in cell lines (NHDF, U-87) and primary cell cultures irradiated with 1–4 Gy gamma-rays. U-Net CNN^64^ was used for nucleus segmentation and foci detection, followed by a Maximally Stable Extremal Region (MSER) algorithm^69^ for segmentation of detected foci. DeepFoci algorithm obtained F1 = 0.67 and worked with 3D confocal multichannel data.

Recently, *Redmon *et al*.* introduced the You-Only-Look-Once (YOLO) approach^70–72^. This uses a real-time object detection algorithm which is fast, simple to construct and can be trained directly on full images. The YOLO algorithm processes the object detection problem as a regression problem, to spatially separate bounding boxes and associated class probabilities. YOLO also generalizes well to new domains, thus making it ideal for applications that rely on robust object detection. Additionally, enhanced versions of the network algorithm operate faster than other detection frameworks^73^. Automated microscopic analysis using YOLO has been proposed for several tasks. These include peripheral leukocyte recognition^74^, cellular detection in fluorescence microscopy^75^, sperm cell detection^76^, parasite stage of malaria-infected red blood cells^77^. To our knowledge, the YOLO algorithm has not been used for automated γ-H2AX foci scoring of human peripheral blood samples.

In this study, we assessed an automated γ-H2AX foci scoring system based on confocal fluorescence microscopic images which were captured from white blood cells (WBCs) irradiated with high doses in the range of 0–6 Gy. We introduced FociRad, a two-stage (or concatenated) approach of the YOLO model, which could localize mononuclear cells (MNCs) and detect γ-H2AX foci inside their nuclei. The objectives of the present research were (1) to develop an automated foci counting method based on concatenated deep-learning (FociRad), (2) to evaluate the performance of FociRad with captured WBC images, (3) to establish dose–response calibration curves, and (4) to implement the FociRad for radiation dose estimation in the selected dataset. All results were compared with those obtained from manual scoring.

## Materials and methods

### Ethics statement

This study was reviewed and approved by the Mahidol University Central Institutional Review Board (Approval No. MU-CIRB 2019/119.1904). In this study, all experiments were performed in accordance with the Declaration of Helsinki.

### Dataset acquisition

#### Blood collection and in vitro irradiation

Blood samples were collected from seven healthy donors (four male and three female volunteers, age 21–25 years) by venipuncture into 5 mL heparinized tubes after informed written consent. Donors were nonsmokers with good health (no obvious illness at time of donation) and no history of exposure to chemotherapy or radiotherapy within the last two years.

Blood samples were aliquoted into 1.5 mL cryovials which were either irradiated with different amounts of X-rays (1, 2, 4 or 6 Gy) or not irradiated and kept as controls (0 Gy). A 3-dimensional radiotherapy plan consisted of 6 MV X-ray, dose rate of 600 MU/min, field size 10 cm^2^, source to surface distance of 95 cm; prescribed doses of 1, 2, 4 or 6 Gy at 5 cm depth were computed on a simulated water phantom using Acuros XB dose calculation algorithm in the Eclipse Treatment Planning System version 16.1 (Varian Medical Systems, Milpitas, CA). Tubes of sampled blood were set up under the treatment plan conditions, placed into the holder at a depth of 5 cm in the water phantom, and irradiated using a TrueBeam linear accelerator (Varian Medical Systems, Palo Alto, CA). In this study, the radiation dose at a depth of interest was determined using a Farmer chamber FC65-G (IBA Dosimetry, Germany), the standard clinical reference dosimeter for high-energy photons.

#### γ-H2AX assay and image acquisition

Irradiated blood samples were incubated at 37 °C for 1 h prior to transferring 500 µL of blood into 15 mL conical bottom tubes for fixation with 500 µL of 8% paraformaldehyde for 10 min. Fixed blood samples were diluted with 1 × PBS to make a final concentration of 1% paraformaldehyde before transporting on ice to a laboratory in the Faculty of Medical Technology, Mahidol University. Blood samples were centrifuged at 1000 rpm at 4 °C for 5 min and incubated in 0.1% (v/v) Triton X-100 diluted in phosphate-buffered saline (PBS) for 30 min. Cells were washed twice with PBS. After removal of red blood cells, WBCs were resuspended in 1 mL 50% methanol and placed on ice for 10 min before blocking in 1% bovine serum albumin (BSA) for 30 min. Cells were stained with Alexa Fluor 488 anti-H2A.X Phosphor (Ser139) (BioLegend, USA) (1:25) for 2 h, APC anti-human CD45 (BioLegend) (1:25) for 30 min and DAPI for 10 min. After incubation, cells were washed three times with PBS to remove excess antibodies, and stored in 1% paraformaldehyde overnight. Cells were carefully spread and mounted on glass slides and then kept at 4 °C prior to analysis.

Images were taken with a confocal laser scanning microscope (Zeiss LSM 800 Airyscan confocal laser scanning microscope, Carl Zeiss, Jena, Germany). Channel mode visualization was done using the 100X objective with oil immersion, resulting in images with a resolution of 1024 × 1024 pixels. Three color channels [DAPI (blue), Alexa Fluor 488 anti-H2A.X Phosphor (Ser139) (green), and APC anti-human CD45 (red)] captured confocal images of nuclei, γ-H2AX foci, and surface of leukocytes, respectively. MNCs were differentiated from polymorphonuclear cells by morphologically distinguishing double-positive DAPI and bright CD45 expression. Z-stack images (5 slices per cell) with a slice interval of 0.6 µm were captured and exported as CZI file format, then converted to TIFF file format using modular image acquisition, processing and analysis software for digital microscopy (ZEN 2.1 software, Carl Zeiss Microscopy GmbH). A total of 965 images with no overlapping of neighboring cells were captured to obtain 700 MNCs irradiated at 0, 1, 2, 4 or 6 Gy. Examples of confocal microscopic images of MNCs from different doses can be found in Supplementary Fig. S1 online.

### FociRad: the concatenated deep learning model

**Figure 1:**
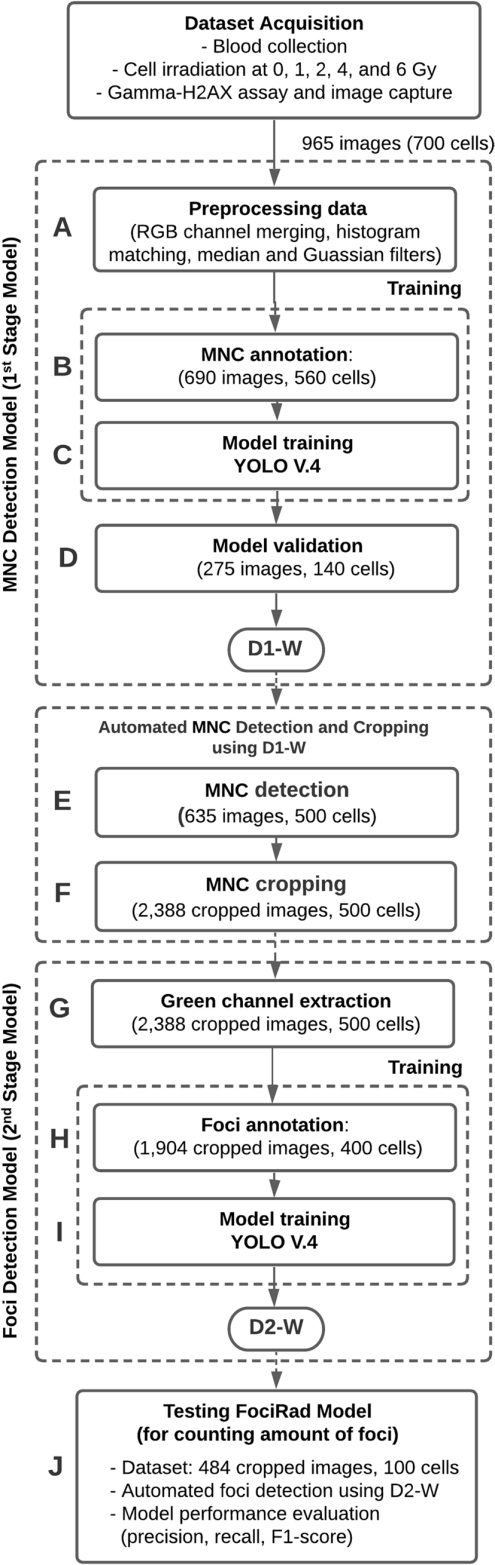
Schematic diagram of the proposed concatenated deep-learning approach (FociRad). Workflow starts from dataset acquisition process. The dataset was imported into the FociRad two-stage detection models, i.e. mononuclear cell (MNC) and foci detection models. D1-weighted for MNC detection (D1-W) was obtained from step A to D. Automated MNC detection and cropping process using D1-W was performed in step E and F. D2-weighted for foci detection (D2-W) was obtained from step G to I. Finally, the performance of FociRad model for counting number of foci using test dataset was evaluated in step J.

The concatenated deep learning approach was proposed as a novel technique that consists of a two-stage deep learning model. The MNC detections in the first-stage model were implemented on captured images using the object detection-based YOLO v4 CNN^73^. Cropped rectangular image regions that were precisely fitted to each observed MNC served as input datasets for the second-stage CNN model. Therefore, there was no two cells visible in a single bounding box. However, with the rectangular bounding box of YOLO v4, there might be a part of the neighboring cells visible at the corner (mostly cell surface and a very small portion of the nucleus). In the second stage, this became a training dataset for the γ-H2AX foci detection model. Figure 1 illustrates the process flow for the proposed method's data preparation and use in model training.

#### Captured image preprocessing (label A in Fig. 1)

**Figure 2:**
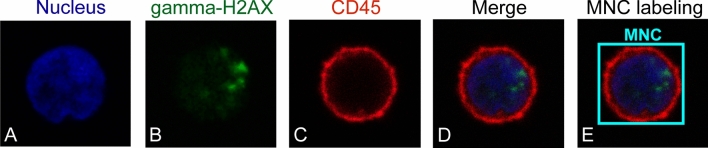
Preprocessing and data labeling used in the first-stage model. Confocal microscopic images of white blood cells. The images showed nucleus in blue (**A**), γ-H2AX foci in green (**B**), and the CD45 surface marker in red (**C**). Those three channels were merged prior to use as a dataset of the cell detection model (**D**). The mononuclear cell (MNC) was manually labelled using a rectangular bounding box (**E**).

Three color channels of the confocal images, which captured nucleus (Fig. 2A), γ-H2AX foci (Fig. 2B), and the CD45 surface marker (Fig. 2C), were merged to generate RGB images (Fig. 2D) using MATLAB R2020a software (MathWorks, USA). During image capture in a typical γ-H2AX assay, there may be variation in intensity between experiments due to the staining and imaging processes. Thus, to eliminate such variation, the standard image processing method [i.e., histogram matching, 3 × 3 median filter, and Gaussian filters (standard deviation of 5)] was employed with all merged RGB images before they were used in the model training process.

#### Mononuclear cell detection model (first-stage model): dataset preparation and training

The purpose of this procedure was to create an object detection model for automatically detecting MNC regions on the captured images. To begin, the dataset was prepared through MNC annotation (label B in Fig. 1). A dataset of 690 images (560 cells) was chosen at random from the group of captured MNC images. MNC annotation was performed by a trained biologist. MNCs were differentiated from polymorphonuclear cells by morphologically distinguishing double-positive DAPI and bright CD45 expression in RGB images as mentioned previously. We used a rectangular bounding box to manually label each MNC on the RGB image and excluded cells near the surface as well as those with irregular shapes (incomplete cells). The bounding box's central point was situated in the center of each MNC. The captured images were manually labelled as “MNC” (Fig. 2E). Manually scored image sets were assembled and used to train the MNC detection model.

**Figure 3:**
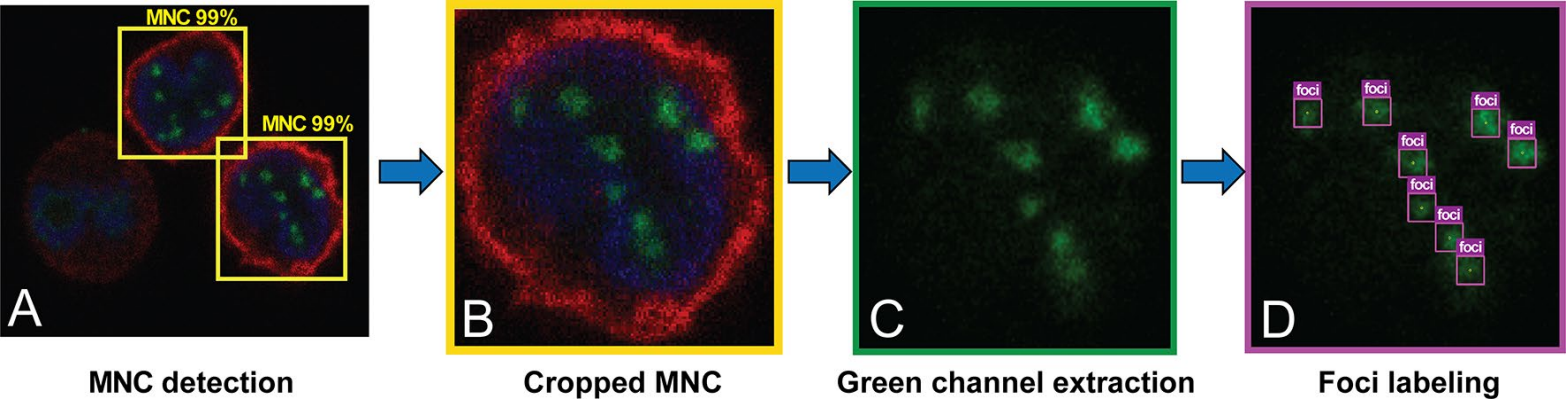
Cell detection, cell cropping, preprocessing data (green channel extraction) and foci annotation process. Mononuclear cell (MNC) detection in a three-channel z-stack image with detection confidence (**A**), a cropped cell (**B**), green channel extraction (γ-H2AX foci) (**C**), and foci labeling (**D**).

The labelled MNC images were imported into an in-house deep-learning model development platform (CiRA CORE, https://web.facebook.com/groups/cira.core.comm/) to train the MNC detection-based CNN model (YOLO v4) (label C in Fig. 1). For training, Intel 1151 ®Core™ i5-9600 K CPU, NVIDIA GeForce RTX 2070 GPU and250 GB RAM were used. The total computation time was 13 h, 29 min and 25 s. MNC detection models were validated with 275 images (140 cells) (label D in Fig. 1). The parameters, denoted as D1-weighted for MNC detection (D1-W), were obtained after the model training by choosing the best performance of the detection. Detection accuracy reached 96.6% (sensitivity 96.7% and specificity 96.5%) for MNC detection. The detected area of each MNC covered the entire bounding box (Fig. 3A).

#### γ-H2AX foci detection model (Second-stage model): dataset preparation and training

The dataset obtained from 635 images (500 cells) was used to develop the γ-H2AX foci detection model (label E in Fig. 1). The trained MNC detection model described as the first-stage model was employed with optimized D1-W parameters. It was used to prepare the cropped MNC images by operating with the captured images as input. The cropping operation was performed on the detected MNC regions (Fig. 3B) to obtain 2388 cropped MNC images (500 cells) (label F in Fig. 1). Model outputs corresponded to the data from the bounding boxes of MNCs. These bounding boxes were used to provide position and area for cropping the MNC images.

Subsequently, extraction of green channel data (label G in Fig. 1) was performed to separate γ-H2AX foci areas (Fig. 3C) using MATLAB R2020b software (MathWorks). The γ-H2AX foci were manually labelled by a trained biologist (label H in Fig. 1). We used a rectangular bounding box to manually label each γ-H2AX foci on the cropped image. These cropped MNC images were manually labelled as “foci” (Fig. 3D). Manually scored image sets were assembled and used to train the model for γ-H2AX foci detection. The training dataset (1,904 cropped MNC images, 400 cells) was imported into our in-house, deep learning framework (CiRA CORE) to train the γ-H2AX foci detection-based CNN model (YOLO v4) (label I in Fig. 1). For training, Intel 1151 ®Core™ i5-9600 K CPU, NVIDIA GeForce RTX 2070 GPU and 250 GB RAM was used. The total computational time was 37 h, 1 min and 57 s. The parameters denoted as D2-weighted (D2-W) were obtained after the model training by choosing the best performance of the foci detection model.

#### γ-H2AX foci detection model testing and performance evaluation

FociRad was employed to test 484 cropped images (100 cells) (label J in Fig. 1). Following that, the D2-W qualified weight was used for automatic γ-H2AX foci recognition. The experiments were carried out on a workstation with Intel 1151 ®Core™ i5-9600 K CPU, NVIDIA GeForce RTX 2070 GPU and 250 GB RAM.

**Figure 4:**
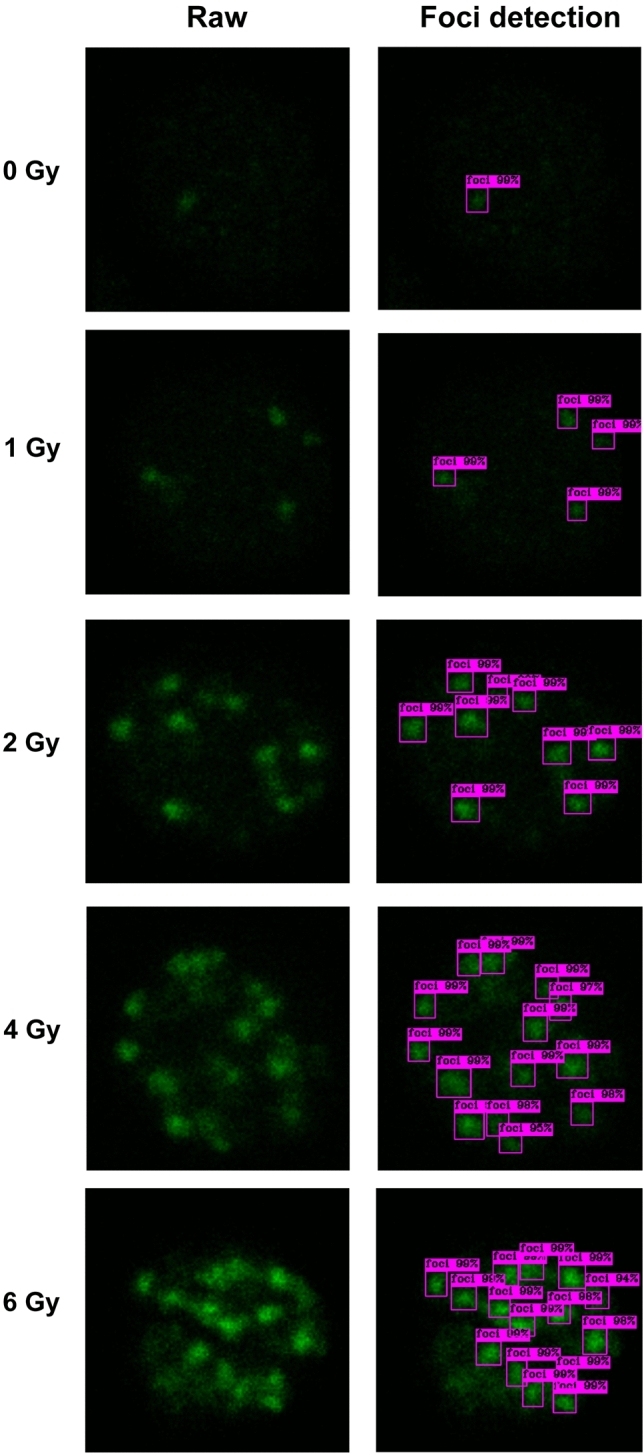
Performance testing of the FociRad. Green channel cropped images of cells irradiated with X-rays (0, 1, 2, 4 or 6 Gy) (left column) and automated foci detection with confidence thresholds by the FociRad model (right column) are shown.

Figure 4 presents examples of automated foci detection using FociRad. The number of γ-H2AX foci in each cropped image was automatically counted and recorded in a Microsoft excel file. The mean number of foci per cell at each dose of irradiation was calculated. A video of the foci detection model being tested was posted on YouTube at https://bit.ly/3zrBhcz.

To assess γ-H2AX foci detection performance, the indexes of precision and recall ratio were calculated as in Eqs. (1) and (2).

1 $$Precision = \frac{TP}{{TP + FP}}$$

2 $$Recall = \frac{TP}{{TP + FN}}$$

where TP is true positive, FP is false positive and FN is false negative. Notably, we did not use accuracy as a measure, as more than 90% of the training pixels were true negatives (TN), making accuracy and specificity insufficient metrics^60^. F1 score is a harmonic average of precision and recall rate, and can be defined as in Eq. (3).

3 $$F1 score = \frac{2 \times Precision \times Recall}{{Precision + Recall}}$$

In the calculation of F1 score, true positive (TP), false positive (FP) and false negative (FN) values were used which has been confirmed by manual scoring.

### Implementation in radiation dose estimation

We implemented the proposed FociRad for radiation dose estimation in an unseen dataset, which was collected from the blood sample of one healthy female donor (age, 21 years), then these dose estimates were compared with those obtained by manual scoring. The unseen dataset contained 80 MNCs irradiated with 0, 2, 4 or 6 Gy (160 captured images). All captured images were preprocessed before manual or automated scoring (see “Captured image preprocessing (label A in Fig. 1)” section).

**Figure 5:**
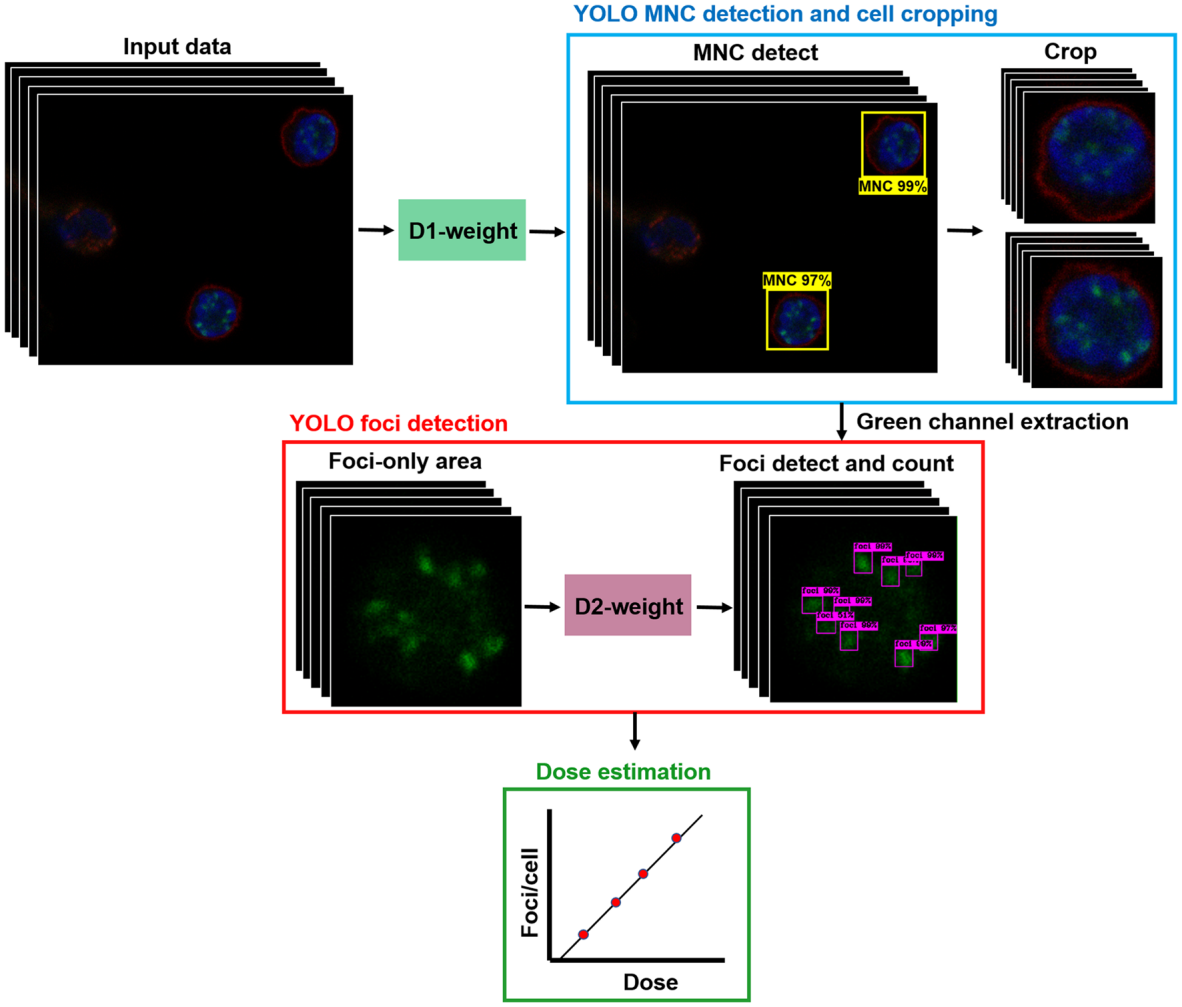
FociRad implementation process. Three-channel z-stack images were analyzed by D1-weighted for mononuclear cell (MNC) recognition and cell cropping. Green channel extraction was performed to show only γ-H2AX foci. Green channel images were analyzed by D2-weighted for foci detection. Foci were automatically counted and the numbers of foci per cell were used to calculate dose by linear fitting to the established dose–response calibration curves.

For manual scoring, two independent observers performed the foci counting. Inter-rater reliability was determined to assess overall agreement between the two observers. The images were fully blinded before analysis and the two observers had no information about radiation dose exposure to the blood samples. Shrout-Fleiss^78^ intraclass correlation coefficient (ICC) estimates were calculated for inter-rater reliability based on a two-way, random effects model with mean-rating (k = 2) and absolute agreement; a two-way mixed-effect model, based on single rating and absolute agreement, was used to assess the intra-rater reliability for each rater. ICCs were calculated using SPSS statistical package, version 18.0 (SPSS, Chicago, IL). ICC was found acceptable by statistical standards (ICC = 0.8)^79^. Dose–response calibration curve from manual scoring was employed for dose estimation. For the automated scoring using the FociRad, preprocessed images were analyzed by the optimized D1-weighted model for MNC detection, followed by the D2-weighted model for γ-H2AX foci detection and foci counting. Dose–response calibration curve established from FociRad was then used for dose calculation (Fig. 5).

Radiation dose estimation in the unseen dataset was performed using the in-house dose–response calibration curves (i.e. the relationship between radiation dose against mean number of γ-H2AX foci per cell). To establish a dose–response calibration curve, mean numbers of foci per cell were scored manually by a trained biologist and automatically by FociRad. The FociRad was employed to establish dose–response calibration curves using the D1-W for MNC detection, and then the D2-W for γ-H2AX foci detection. Mean numbers of γ-H2AX foci per cell from manual scoring and the FociRad were fitted individually using a linear function. SPSS statistical package, version 18.0 (SPSS, Chicago, IL) was used for curve fitting. The established dose–response calibration curves for both manual and automated scoring were linear and fitted with the equation y = ax + b where y was the mean number of γ-H2AX foci per cell and x was the absorbed dose (Gy). Dose–response calibration curves established from both manual and automated FociRad scorings were employed for converting foci counts into dose estimates.

Estimated doses obtained from both methods were used to calculate mean absolute difference (MAD) values^80^ as in Eq. (4).

4 $$MAD = \left| {Estimated\;dose\ {-}\ actual\;dose} \right|$$

## Results

### Model performance metric results

The performance evaluation metrics included precision, recall, and F1 score. These metrics of the γ-H2AX foci detection (FociRad) model for irradiated doses of 0, 1, 2, 4 and 6 Gy were presented in Table 1. Overall model performance metrics were 0.924, 0.897, and 0.911 for precision, recall and F1 score, respectively.

**Table 1:** γ-H2AX foci detection model evaluation. Performance metrics by dose for precision, recall and F1 score.

Metrics	0 Gy	1 Gy	2 Gy	4 Gy	6 Gy	Overall
Precision	0.889	0.962	0.920	0.931	0.910	0.924
Recall	0.667	0.906	0.947	0.877	0.886	0.897
F1 score	0.762	0.933	0.933	0.903	0.898	0.911

### Agreement of γ-H2AX foci assessments between manual and automated (FociRad) scoring

γ-H2AX foci per cell determined by manual scoring compared with FociRad scoring were assessed with intra-class correlation coefficients (ICCs)^78^ using SPSS version 18.0 (SPSS, Chicago, IL). Bland–Altman analysis^81^ was used to visually determine systemic differences, and estimate their means and limits of agreement (LOA), between manual and automated scorings. Bland–Altman plots, which presented the differences between the two methods versus the mean values from manual scoring with the representation of the LOA (from –1.96 SD to + 1.96 SD), were constructed and evaluated. For determining the extent of agreement between the two methods, ICC estimates^78^ and their 95% confidence intervals (CIs) were calculated based on absolute-agreement and two-way random effects models. Statistical analyses were performed and plots created with SPSS version 18.0. Mean numbers of γ-H2AX foci obtained from the FociRad showed very good agreement [ICC = 0.970 (0.962–0.977)] with those from manual scoring (see Supplementary Fig. S2A online); mean foci difference (mean number of foci_Human_ − foci_FociRad_) was – 0.001 ± 0.874 foci (see Supplementary Fig. S2B online).

### Comparison of estimated doses based on manual and automated (FociRad) foci scoring

The dose–response relationship of manual scoring in the range of 0–4 Gy was y = 1.996x + 0.769 (R^2^ = 0.968), while FociRad was y = 1.739x + 1.080 (R^2^ = 0.913). The uncertainties were presented as standard errors (see Supplementary Table S1 online). Intra-class correlation coefficient (with 95% CI) for inter-rater reliability of γ-H2AX foci manual scoring between two observers was 0.990 (0.986–0.992). Intra-rater reliability was 0.981 (0.977–0.985) and 0.968 (0.958–0.975) for observers 1 and 2, respectively. Table 2 presents the estimated doses and MAD values, which were compared between those estimated from manual scoring and FociRad.

**Table 2:** Estimated doses and MAD values obtained from manual and FociRad scoring.

Actual dose	0 Gy		2 Gy		4 Gy		6 Gy		Average MAD
Estimated dose	Gy	MAD	Gy	MAD	Gy	MAD	Gy	MAD	
Manual	− 0.27	0.27	2.75	0.75	4.31	0.31	5.11	0.89	0.56
FociRad	− 0.52	0.52	2.93	0.93	4.67	0.67	5.64	0.36	0.62

MAD = mean absolute difference.

## Discussion

The intensity-based approach to quantitate γ-H2AX for dose estimation is a more rapid and reliable method for detecting DNA damage within the first few hours of radiation exposure compared to other cytogenetic methods which require several days. Still, manual scoring of γ-H2AX foci needs an extensive time for scoring and is labor intensive. Therefore, we proposed an automated foci scoring method (FociRad) using a concatenated deep-learning model to overcome these limitations and compared performance with manual scoring.

Several groups^32,59,60^ successfully developed deep learning methods in automated foci scoring in different types of cells and yielded very good performances as mentioned in the introduction section. Moreover, Vicar et al.^68^ studied using 3D data and in heterogeneous cell populations. Previous studies^32,59,60,68^ neither used YOLO algorithm for object detection nor studied in human blood samples. FociRad, the two-stage learning method of the YOLO v4 for MNC and γ-H2AX foci detection, was applied in whole blood and yielded a very good F1 score of 0.911 (min 0.762, max 0.933). Moreover, automated scoring had very good correlation with manual scoring at 0, 1, 2 Gy (ICC = 0.90–0.94), and good correlation at 4 Gy (ICC = 0.80) and 6 Gy (ICC = 0.86). At higher doses, the dispersion of data between automated and manual scoring may have been due to the difficulties in differentiating overlapped foci. This is observed at high doses^36,50^ and limits the γ-H2AX assay using fluorescence microscopy. To address this limitation, the adaptive FociRad model using deep learning-based clustering approaches^82^ should be studied in three-dimensional confocal images in the future.

In radiation emergency, biodosimetry network played an important role in radiation dose estimation. A previous study by Rothkamm et al.^80^ showed that the MAD values between estimated and actual doses based on the scoring of foci by four participating laboratories could be categorized into two groups: low MAD (0.5 to 0.7 Gy) and high MAD (1.3 to 1.7 Gy). In this study, FociRad model was implemented to estimate the radiation dose from an unseen dataset compared to manual scoring using the established dose–response calibration curves. The MAD values of 0, 2, 4 and 6 Gy actual dose were within the differences of ± 1 Gy for both scoring methods. Our average MAD values were within their low MAD group from the estimation using both manual (0.56 Gy) and FociRad (0.62 Gy) scoring.

FociRad is a fast-processing approach compared with manual scoring. The evaluation times (per 100 cropped images) used by two observers for manual scoring and used by FociRad for automated scoring of the unseen dataset were assessed (see Supplementary Table S2 online). FociRad used consistently shorter times (less than 0.5 min), while manual scoring used longer times which varied with observer. In addition, manual scoring of γ-H2AX foci tended to consume more evaluation time at higher doses as seen from average evaluation times of sevenfold, tenfold and 14-fold at 2, 4 and 6 Gy compared to that at 0 Gy, respectively. This was likely due to the number of overlapped foci in high dose images. Fast evaluation time is important for the application of FociRad in large scale accidents, as potential biodosimetry tools must rapidly estimate dose prior to further medical care. Increasing need of well-established biodosimetry laboratories is important for radiation emergency preparedness. An example is the inter-laboratory comparison exercises conducted by established biodosimetry laboratories as in the European Network of Biodosimetry (RENEB)^83,84^. However, some discrepancies arise between dose estimations from different laboratories^80,83,84^ possibly due to variation in staining and imaging set ups as well as observer-dependent scoring. Using a single foci counting-based, deep learning platform such as FociRad may reduce these inconsistencies.

This study has some limitations, and so further studies will be required. First, the calibration curves were established based on a single timepoint (1 h post-irradiation). However, this time point was within the window of peak γ-H2AX responses (between 30 min and 2 h)^11,85^. Following the induction of γ-H2AX, both a fast and slow decay of the γ-H2AX signal is observed within 24 h and up to a few days^24,86^. It is suggested that calibration curves be established at various time points for wider application. Second, the cell surface marker used in this study (i.e. CD45) is a leukocyte common marker which is not specific to a WBC subpopulation. For our analysis we selected MNCs (lymphocytes and monocytes) rather than polymorphonuclear cells (granulocytes) due to the higher expression of radiation-induced γ-H2AX in these cells^85,87^. Nonetheless, using CD45 as a surface marker may have helped FociRad increase the accuracy of cell recognition in the first-stage model. Moreover, FociRad might also be helpful in quantification of DNA damage resulting from anti-CD45 targeted radioimmunotherapy^88–90^. To further develop the FociRad model for γ-H2AX assessment in different blood cell populations, one could modify the model by staining with other specific surface markers (e.g. T cell – CD3, B cell – CD19, NK cell – CD56, monocyte – CD14, granulocyte – CD15). Finally, FociRad should be further implemented with available external datasets from various sources of radiation (e.g. gamma rays, alpha particles and neutrons), and different energy ranges to reflect those used in diagnostic radiology, nuclear medicine and radiotherapy.

## Conclusion

An automated γ-H2AX foci scoring model (FociRad), with a novel two-stage deep-learning approach based on a YOLO algorithm, was proposed to overcome the limitations of manual scoring of γ-H2AX foci in peripheral blood samples. FociRad functioned with very high performance (F1 > 0.9) and rapid evaluation time (less than 0.5 min per 100 images), when compared to manual scoring which is tedious, observer-dependent and time-consuming. FociRad may be used for rapid and large-scale analysis of γ-H2AX foci, such as is needed for medical triage after a radiation incident.

## Supplementary Information

Supplementary Information.

## Data Availability

The data that support the findings of this study are available at https://github.com/Khaisang/FociRad.git. Furthermore, CiRA CORE can be requested and downloaded on https://git.cira-lab.com/cira.
