# Supplementary material for: A deep learning model (FociRad) for automated detection of γ-H2AX foci and radiation dose estimation
Source: Sci Rep. 2022 Apr 1;12:5527. doi: 10.1038/s41598-022-09180-2 (PMC8975967; doi:10.1038/s41598-022-09180-2)
Supplement: Supplementary file 1 — Supplementary Information. [file 41598_2022_9180_MOESM1_ESM.pdf]

Supplementary Information for:

A Deep Learning Model (FociRad) for Automated Detection of γ-H2AX Foci and Radiation Dose Estimation

Rujira Wanotayan[1], Khaisang Chousangsuntorn[1], Phasit Petisiwaveth[1], Thunchanok Anuttra[1], Waritsara Lertchanyaphan[1], Tanwiwat Jaikuna[2], Kulachart Jangpatarapongsa[3], Pimpon Uttayarat[4], Teerawat Tongloyn[5], Chousak Chousangsuntorn[6], and Siridech Boonsaeng*[6]

***Correspondence to**

Siridech Boonsaeng Email: siridech.bo@kmitl.ac.th

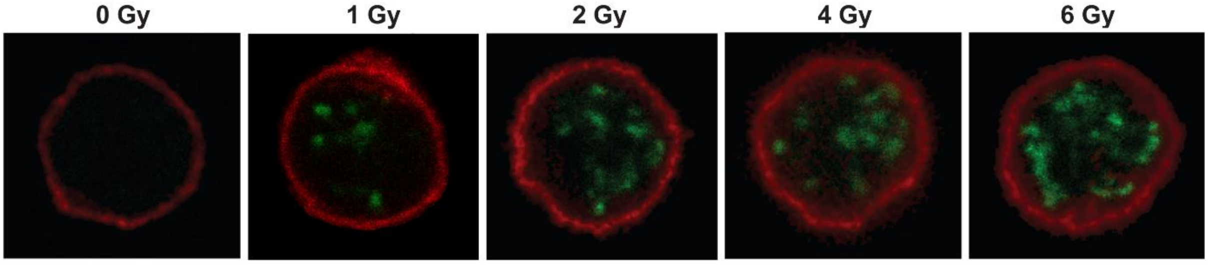

Figure S1 Two-channel z-stack images of cells irradiated with X-ray (1, 2, 4 and 6 Gy) and unirradiated control reveal γ-H2AX foci (green) in cell and CD45 surface marker (red). The number of γ-H2AX foci increased depending on the radiation dose, whereas no change in CD45 expression was observed.

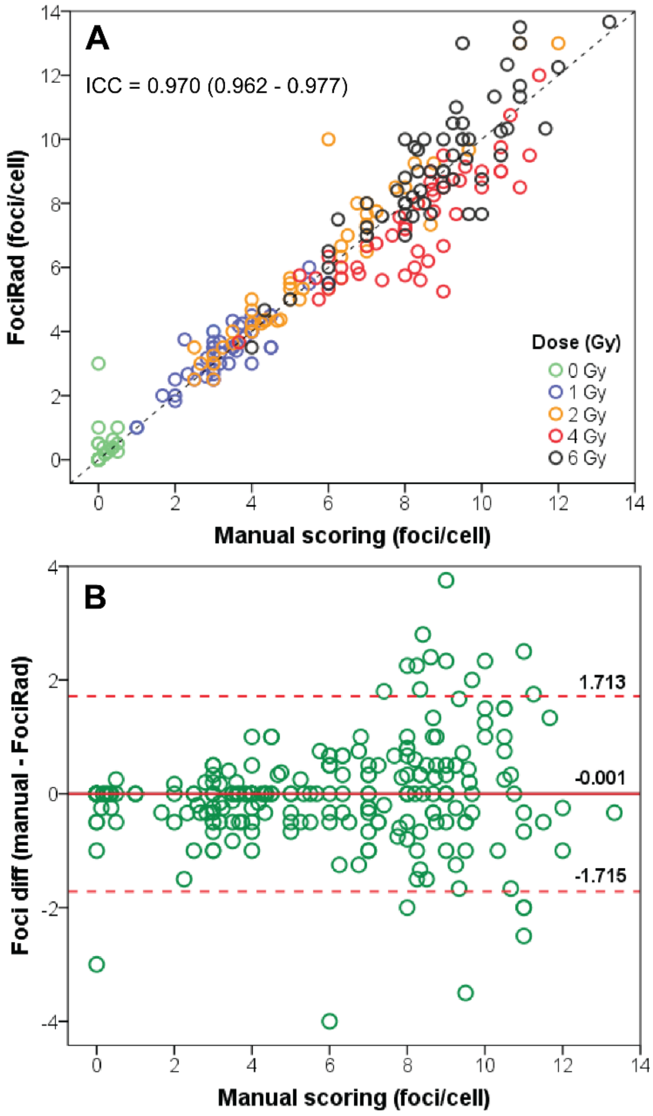

Figure S2 Scatter diagrams of mean number of foci per cell between manual scoring vs. automated scoring (FociRad) and ICCs with 95% CI (A). Bland-Altman plots with data points for each MNC. The y-axes show the difference (diff) in the values of mean number of foci per cell between manual scoring and the FociRad. The x-axes show the mean number of foci per cell from the manual scoring. The red solid line indicates the mean difference (value and percentage of the value expressed on the red solid line). The dashed lines indicate the LOA (B).

Table S1 Calibration curve coefficients (± standard errors).

Methods	a	b
Manual scoring	1.996±0.257	0.769±0.589
FociRad	1.739±0.378	1.080±0.867

Linear equation: y = ax + b   a : linear coefficient   b : constant

Table S2 Evaluation times per 100 cropped images of the unseen dataset at each exposed radiation dose. Manual scoring evaluated by two independent observers, and automated scoring evaluated by FociRad.

Dose (Gy)	Evaluation time (s)			
	Observer 1	Observer 2	Manual scoring	FociRad
0	346 ± 11.3	539 ± 75.7	442 ± 136	27.3
2	3482 ± 182	2532 ± 308	3007 ± 671	27.9
4	4982 ± 1401	4141 ± 907	4562 ± 594	27.5
6	6767 ± 2808	5724 ± 2526	6246 ± 737	27.3
